# Caries prevalence and tooth loss in Hungarian adult population: results of a national survey

**DOI:** 10.1186/1471-2458-8-364

**Published:** 2008-10-21

**Authors:** Melinda Madléna, Péter Hermann, Marianna Jáhn, Pál Fejérdy

**Affiliations:** 1 Department of Pediatric Dentistry and Orthodontics, Faculty of Dentistry, Semmelweis University, Budapest, Hungary; 2 Department of Prosthetic Dentistry, Faculty of Dentistry, Semmelweis University, Budapest, Hungary

## Abstract

**Background:**

Oral health is basicly important for the well-being of people. Thus, it is strongly suggested to organize epidemiological surveys in order to gain representative data on oral condition of the given population. The purpose of the cross-sectional study was to determine the results on tooth loss and caries prevalence of Hungarian adults in different age groups.

**Methods:**

Altogether 4606 persons (2923 women, 1683 men) participated in the study who were classified into different age groups: 19 [less than or equal to], 20–24, 35–44, 45–64, 65–74, [greater than or equal to]75 year olds. Probands were selected randomly from the population attending the compulsory lung screening examinations. The participants were examined by calibrated dentists, according to the WHO (1997) criteria. In order to produce representative data, the chosen localities for these examinations covered the capital, the largest towns, the villages, and case weights were used for the statistical evaluation.

**Results:**

The mean values of DMF-T were found between 11.79±5.68 and 21.90±7.61 These values were significantly higher in women compared to men (p < 0.05). In all age groups the values of M were the highest. Except for the women in the groups of 35–44 and 45–64 year olds, these values showed an increasing tendency both in women and men by age (from 5.50±6.49, and 4.70±4.08 to 21.52±9.07 and 18.41±8.89 respectively). The values of D components reached the highest values in 45–64 year olds (4.54±2.12 and 4.22±2.81, by gender, respectively), then in the older age groups there was a high reduction in these values (in 65–74 year olds: 2.72±1.88 and 1.36±2.48; in 75 or more than 75 year olds: 1.05±1.41 and 1.03±1.76 by gender, respectively). The ratio of D and F values was the highest in the age group of 65–74 year olds (2.12), the lowest ratio could be calculated in 20–34 year olds (0.65).

Data showed some decrease in caries experience in 35–44 years of age between 2000 and 2004. The prevalence of persons with 21 or more teeth had been increased from 65.6% to 73.1%. This positive tendency has not been occured in prevalence of edentulousness in this age group: the prevalence of edentulous persons changed from 1.4 to 1.9%. In 65–74 year olds the level of edentulousness became lower, from 25.9 to 14.8% and the prevalence of persons with 21 or more teeth is higher (22.6%) than it was in 2000 (13.0%).

**Conclusion:**

Present data from Hungary show some slight decrease in caries experience between 35–44 years of age, although this positive tendency has not been occured in prevalence of edentulousness in this age group. A positive tendency could be experienced in the group of 65–74 year olds in edentulousness and in number of teeth, but further efforts are needed to reach a better situation.

## Background

Oral health means not only healthy teeth but it is an integrated part of general health, and basicly important for the well-being of people. Thus, it is strongly suggested to organize epidemiological surveys in order to gain representative data on oral health of the given population. The data gained by different epidemiological examinations should be compared with each other internationally, and also with the previous national records to monitor the changes in dental condition.

In Hungary dental epidemiological examinations of adult population organized by WHO were carried out first in 1985, then it was followed in 1991 [1]. The latest survey was performed in 2000, in two Hungarian age groups [2].

The aims of this study were to gain exact, comparable data on dental situation and of the Hungarian adult population, to evaluate the loss of teeth and caries experience performed during a certain period of time, between 2000 and 2004, in different age groups from 19 ≤ to 75 ≤ year olds.

## Methods

The epidemiological study was organized between 1^st ^of November 2003 and 30^th ^of April 2004, by the Department of Prosthetic Dentistry, Semmelweis University. The consent of the appropriate ethical committee (TUKEB, Semmelweis University, Budapest) was obtained prior to the start of the study. Subjects were volunteers, who had given informed, written consent.

The examinations were carried out by seven calibrated dentists. Depending on the size of the examined population samples, the number of examiners ranged between three and five. The intra- and inter- examiner calibrations were performed according to WHO standards at the begining of the study [3]. All examiners were trained by an experienced person. It was followed by practical training when adults with different ages were examined by each of the examiners. The diagnosis found by the calibrated dentists was compared to those recorded by the reference examiner. The calculation of interexaminer agreement yielded a mean kappa value of 0.85.

Altogether 4606 persons (2923 women, 1683 men) were examined (Table 1). The age of the examined subjects was between 17 and 102 years. Probands were selected randomly from the population attending compulsory lung screening examinations in different sites of the country. After a written consent there were no inclusion or exclusion criteria. There were only some people (19 persons) who didn't want to participate, because they had no enough time. After the examinations there was no any drop out. In order to produce really representative data at national level, case weight was used when calculating proportions, averages and other statistics [4]. By the use of weights the whole sample became representative for the country by sex and age.

**Table 1:** Persons participating in the national survey

**Age groups (years)**	**Gender**		**Total population**
		
	**Male**	**Female**	
19≤	75	38	113

20–34	418	548	966

35–44	259	473	732

45–64	647	1298	1945

65–74	196	416	612

≤ 75	88	150	238

All	1683	2923	4606

The clinical examinations were performed in the place of compulsory lung screening examinations (free rooms of the local government) with plane dental mirror and probe using arteficial light according to the standard methods and criteria of the World Health Organization [3]. Diagnostical criteria and codes for recording the dental condition were as follows:

A crown was recorded as sound if it shows no evidence of treated or untreated clinical caries.

Decayed crown: caries was recorded when a lesion can be seen and feel with the probe in a pit and fissure or on a smooth surface.

Filled crown with decay: when the crown has one or more permanent restorations and one or more areas that are decayed.

Filled crown with no decay: when one or more permanent restorations are present and there is no caries anywhere on the crown. A tooth that has been crowned because of previous decay is recorded in this category.

Missing tooth as a result of caries: it is used for the teeth that have been extracted because of caries and is recorded under coronal status.

Permanent tooth missing for any reason: it is used for permanent teeth judged to be absent congenitally or extracted for orthodontic reason or periodontal disease, trauma etc. Teeth scored as missing for any reason are excluded from all calculations concerning dental caries.

Unerupted crown: it is used only for a tooth space with an unerupted permanent teeth but without primary tooth. Teeth scored as unerupted are excluded from all calculations concerning dental caries.

Radiographs were not taken. The data were coded by the calibrated dentists on a special computerized survey sheets by the computer in place.

Caries prevalence was evaluated later based on DMF-T mean values by gender and different age groups, which were as follows: ≤19, 20–24, 35–44, 45–64, 65–74, 75≤ year olds. Simultaneously, examinations were carried out assessing other conditions (periodontal diseases, oral hygienic habits, dental surgeon attendance etc.), reported elsewhere.

Statistical analysis was performed applying descriptive statistics and the independent sample T test, using Statistical Package for Social Sciences (SPSS) for Windows, 13.0 program package.

## Results

Table 2 shows the distribution of the number of teeth present in different age groups of adults in Hungary. Compared the age group of 35–44 year olds to 65–74 years old group, the degree of edentulousness showed a remarkable increasing tendency with age in last two categories (Table 2).

**Table 2:** Distribution of number of teeth present in different age groups of adults in Hungary (% of persons)

**Number of teeth**	**Age groups (years)**					
	
	**≤19**	**20–34**	**35–44**	**45–64**	**65–74**	**75≤**
21 or more teeth	95.0%	86.3%	73.1%	43.9%	22.6%	9.7%

Less than 21 teeth	0.6%	7.9%	18.1%	23.8%	17.9%	13.7%

*Less than 15*	4.4%	5.2%	6.9%	*26.1%*	*39.7%*	37.9%

*Edentulous*	0.0%	0.6%	1.9%	*6.2%*	*19.8%*	38.7%

Table 3 shows the trends in distribution of number of teeth present in age groups of 35–44 and 65–74 year olds comparing to the previous published data (Czukor, 1994; Szőke and Petersen, 2004 – data from 2000!) [1,2]. (Table 3). In the groups of 35–44 year olds, the percentage of edentulous persons showed continuous increasing tendency, while there was a remarkable decrease in edentulousness in 65–74 year olds between 1991 and 2004. Comparing the previous data, originated from 2000, to present data, there are more people having 21 or more present teeth in the age group of 65–74 year olds in 2004.

**Table 3:** Trends in distribution of number of teeth present in age groups of 35–44 and 65–74 year olds (% of persons)

**Number of teeth**	**Year of examinations**							
	1985*		1991**		2000***		**2004**	
	
	**35–44 year olds**	**65–74 year olds**	**35–44 year olds**	**65–74 year olds**	**35–44 year olds**	**65–74 year olds**	**35–44 year olds**	**65–74 year olds**

21 or more teeth	70.3	No data	No data	No data	65.6	13.0	73.1	22.6

Less than 21 teeth	15.3	No data	No data	No data	22.6	17.3	18.1	17.9

Less than 15	13.8	No data	No data	No data	10.3	43.8	6.9	39.7

Edentulous	0.3	No data (1986: 18.0)	1.2	53.3	1.4	25.9	1.9	19.8

* Czukor (1994) [1]

** Czukor (1994) [1]

***Szőke and Petersen (2004) [2]

In the examined population the caries prevalence was significantly higher in women compared to men in all age groups (p < 0.05) (Table 4). In all age groups the values of *M *were the highest. Except for the women in the groups of 35–44 and 45–64 year olds, these values showed an increasing tendency both in women and men by age (from 5.50 ± 6.49, and 4.70 ± 4.08 to 21.52 ± 9.07 and 18.41 ± 8.89 respectively). The values of *D *components reached the highest values in 45–64 year olds (4.54 ± 2.12 and 4.22 ± 2.81 by gender, respectively), then in the older age groups there is a high reduction in these values (in 65–74 year olds: 2.72 ± 1.88 and 1.36 ± 2.48; in 75 or more than 75 year olds: 1.05 ± 1.41 and 1.03 ± 1.76 by gender, respectively). The ratio of D and F values was the highest in the age group of 65–74 year olds (2.12), the lowest ratio can be calculated in 20–34 year olds (0.65).

**Table 4:** Caries prevalence and its components in the Hungarian adult population (mean ± S.D.)

**Age groups (years)**	**Participants/Gender**	DMFT*	**D**	**M**	**F**
19≤	Male	11.24 ± 4.85	4.20 ± 3.39	4.70 ± 4.08	2.10 ± 2.08
	
	Female	12.34 ± 7.14	2.81 ± 2.93	5.50 ± 6.49	4.27 ± 3.55
	
	All	*11.79 ± 5.68*	*3.50 ± 3.21*	*5.10 ± 5.05*	*3.19 ± 3.06*

20–34	Male	12.28 ± 6.19	2.90 ± 3.21	5.73 ± 6.07	3.65 ± 3.96
	
	Female	13.25 ± 5.65	2.48 ± 3.11	6.09 ± 5.78	4.68 ± 4.09
	
	All	*12.76 ± 5.45*	*2.69 ± 2.36*	*5.91 ± 4.93*	*4.16 ± 4.20*

35–44	Male	14.73 ± 5.88	4.12 ± 3.18	8.51 ± 6.16	2.10 ± 4.00
	
	Female	16.07 ± 5.16	4.48 ± 2.52	9.29 ± 6.02	2.30 ± 4.47
	
	All	*15.40 ± 5.13*	*4.30 ± 1.91*	*8.90 ± 4.66*	*2.20 ± 4.32*

45–64	Male	14.07 ± 7.56	4.22 ± 2.81	9.05 ± 8.54	0.80 ± 3.16
	
	Female	17.03 ± 6.87	4.54 ± 2.12	9.09 ± 8.52	3.40 ± 3.50
	
	All	*15.55 ± 7.00*	*4.38 ± 1.51*	*9.07 ± 7.24*	*2.10 ± 3.37*

65–74	Male	18.99 ± 8.45	1.36 ± 2.48	16.59 ± 9.52	1.04 ± 2.07
	
	Female	24.81 ± 8.50	2.72 ± 1.88	21.21 ± 9.70	0.88 ± 1.94
	
	All	*21.90 ± 9.00*	*2.04 ± 1.45*	*18.90 ± 9.29*	*0.96 ± 2.14*

75≤	Male	21.35 ± 7.09	1.03 ± 1.76	18.41 ± 8.89	0.91 ± 1.79
	
	Female	22.44 ± 7.86	1.05 ± 1.41	21.52 ± 9.07	0.87 ± 1.58
	
	All	*21.90 ± 7.61*	*1.04 ± 1.53*	*19.97 ± 9.02*	*0.89 ± 1.65*

*p < 0.05 (between genders)

## Discussion

The level of edentulism is a good indicator of populations' oral health. The main causes may be not only a carious process, but any other reasons, mainly periodontal diseases. According to cariological data, including edentulism, the most important comparable age groups are represented by the groups of 35–44 and 65–74 year olds. However there are only few data on the prevalence of edentulousness resulted from caries of younger age group in different countries. At national level analysing and comparing the data of the present study it can be seen an unfavourable situation in Hungary: the percentage of edentulous persons has been increased continuously in the group of 35–44 year olds (Table 3). Data from France in 1997 shows extremely better results than those of Hungarian data in this age group: none of the examined persons was edentulous and 97% of the sample had more than 20 natural teeth present, but there is no any information about the changes [5].

In 65–74 year olds the percentage of edentulousness shows decreasing tendency according to the data from 1991 (53.3%) and 2000 (25.9%), in 2004 it is 19.8% in Hungary. In groups of elderly the ratio of edentulousness become lower and the number of persons with 21 or more teeth become higher. It can demonstrate those generally published fact that the total number of elderly people with natural teeth has been increased in many countries and this increase is likely to accelerate over this decade [6].

Among Spanish adults aged 65 and over 34% of persons are edentulous, which is a higher ratio, than those of present Hungarian examination's results [7]. That means a relative „good position” for Hungary, by the latest data. By the study of Hugoson et al. in Jönkoping, Sweden, the number of edentulous individuals in thee age groups of 40–70 years was reduced from 16 percent in 1973 to 8 percent in 1993 and to 1 percent in 2003. The mean number of teeth increased in this population [8]. By the data published in 1998 the proportion of 65–74 years old European adults, who are edentulous, varies from 12.8% to 69.6% between 1986–96. [9]. According to the published data after 2000, the percentage of edentulous persons of European elderly are reported from 15% to 46% [10]. Considering its unfavourable economical circustances, it is very interesting that the elderly Lithuanians were found to have lower levels of edentulousness (ranged 11–15%) than elderly people in most of European countries [11].

During the latest years, a considerable caries decline has been observed not only in children, adolescents and young adults, but in the adult population also, mainly in the Western European countries [12-14]. Unfortunately there are only few comparable data showing the trends of changes in DMF-T levels of adults in different countries because of the different methods, age groups, etc, although without proper data on the prevalence and distribution of dental caries, there is little point in setting out goals or strategies either at international or national level.

**Figure 1:**
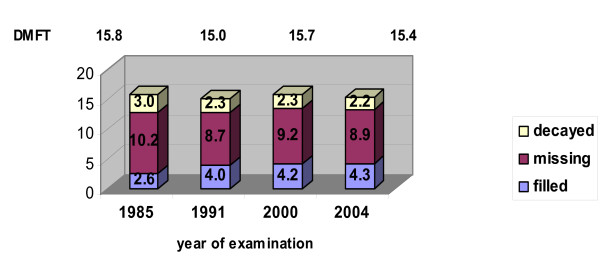
Trends in caries prevalence in age groups of 35–44 year olds between 1985–2004 in Hungary.

Between 1985 and 1991 in the group of 35–44 year olds Hungary shows a decreasing tendency in the prevalence of caries, then it was followed by an increase (between 1991 and 2000) (Figure 1). During the 30 year period (1973–2003), the number of carious lesions and restorations decreased in general in Jönkoping, Sweden [8]. In Slovenia, data show substantial and continued decline of caries prevalence in the adult population [15]. A decreasing tendency has been found in DMFT values in Malta between 1985 and 1990, from 12.7 to 10.0 and Slovenia between 1987 and 1998, from 20.5 to 14.7 in 35–44 year olds [10]. Eriksen (1995) published the average of DMF-T experience in 35 years old adults from Norway, where the DMF-T reduction was 28%, between 1973 and 1993 [16]. In the age group of 35–44 year olds, by the data of european surveys from 1988 the values of DMFT are 19.0, 16.7 and 17.4 (England and Wales, Germany and Netherlands) [17]. In Germany the mean DMF-T values are found to be 16.1 in the 35–44 year olds by Schiffner and Reich (1999) [18]. From the eastern countries data show relative high differences in DMFT values which are between 12 to 19.5 in 35–44 year olds [19,20]. From the components the values of M are the highest, as it can be seen in the present study also (Table 4). In Hungary, comparing the latest data to those of previous WHO examination's results, the number of filled teeth had been increased, while the number of decayed teeth had been decreased between 1985 and 2004 (Figures 1). The values of missing teeth changed down between 1985 and 1991 and had been increased by 2000. The cariological data of adult population didn't show important changes after 2000, the values of M component showed a slight decreasing tendency.

Between 2000 and 2004 in groups of more than 65 year olds, data showed beneficial changes in Hungary. Present Hungarian data can represent further decrease, mainly in values of M component: from 21.32 to 19.97. The trends towards preserving more natural teeth with increasing age, was found in the study of Kovac-Kavcic and Skaleric (2001), matches the trends observed in 30-to 50-year-old Dutch people, in a 40 to 80-year-old population in Sweden [21-23]. Based on the review of Bourgeois et al (1998) the DMF-T index ranged from 22.2 to 30.2 among noninstitutional European adults aged 65–74 years for the period 1986–1996 [9]. In Germany the mean DMF-T values were found to be 23.6 in the 65–74 year-old group by Schiffner and Reich (1999) [18]. There are no further information on changes these data.

The observed differences in caries prevalence of the adult population suggest that it may be possible to develop and implement oral health policies taking into account geographical and socioeconomical differences in populations. According to the results of a dental questionnaire, people cleaned their teeth mostly twice a day using mainly toothbrush and dentifrice, only 12% of them used mouthwash, 11% toothpick and 7% dental floss in Hungary. The majority of the asked population 68% visited dental service on an irregular base (only in case of acute complaints) [24]. Comparing the data to those of other similar studies' data from UK, Netherlands and Sweden, the results show a much more higher level of dental health education of the populations in these countries (eg. 60–95% of adults visit the dentists regularly) [13,23,25].

## Conclusion

Present data from Hungary show some decrease in caries experience in 35–44 years of age between 2000 and 2004: the prevalence of persons with 21 or more teeth increased from 65.6% to 73.1%. This positive tendency has not been occured in prevalence of edentulousness in this age group: the prevalence of edentulous persons changed from 1.4 to 1.9%. In 65–74 year olds the level of edentulousness became lower, from 25.9 to 14.8% and the prevalence of persons with 21 or more teeth is higher (22.6%) than it was in 2000 (13.0%). Comparing the changes to the international results, further efforts are needed to reach a better position. These data and comparisons could be used as a base information from a period of time for health authorities and dental profession for planning new strategies, giving a possibility to compare the data after a certain period.

## Competing interests

The authors declare that they have no competing interests.

## Authors' contributions

MM participated in the design and coordination of the study, carried out all statistical analysis and drafted the manuscript. PH participated in the design and the screening. MJ participated in the sreening. PF participated in the design, coordination and supervision of the study. All authors read and approved the final manuscript.

## Pre-publication history

The pre-publication history for this paper can be accessed here:


